# Arachidonic Acid Metabolism Down-Regulation-Mediated Tumor Necrosis Factor Signaling Contributes to Cutaneous Fibrosis and Skull Hyperplasia in Goldfish Hoods

**DOI:** 10.34133/research.0786

**Published:** 2025-08-06

**Authors:** Liang-Liang Li, Qi-Lin Xu, Wen-Jing Yi, Di-Di Ma, Hui Jin, De-Zheng Fu, Xiao-Li Yang, Yang Wang, Zhi Li, Zhong-Wei Wang, Xi-Yin Li, Meng Lu, Xiao-Juan Zhang, Li Zhou, Jian-Fang Gui, Peng Yu

**Affiliations:** ^1^ State key Laboratory of Breeding Biotechnology and Sustainable Aquaculture, HubeiHongshan Laboratory, Innovation Academy of Seed Design, Institute of Hydrobiology, Chinese Academy of Sciences, Wuhan, China.; ^2^ University of Chinese Academy of Sciences, Beijing, China.

## Abstract

Goldfish (*Carassius auratus*) are renowned as a premier ornamental fish in the world. Especially, the hood, a distinctive cephalic skin protrusion, is a highly sought-after feature for its endearing aesthetics. Despite a longstanding hypothesis that the hood is a type of tumor, the details of their composition, structure, and the mechanism of its formation have remained enigmatic. In this study, we attempted to demystify the morphogenetic mechanism of hood development by providing a detailed analysis of the hood’s architectural and compositional attributes, complemented by multi-omics changes across its developmental stages. Our results were also validated through dual-luciferase reporter assays and cytological evaluations in vitro and in vivo. We uncovered a 4-layered complex structure (stratum compactum, stratum spongiosum, stratum adventitia, and epithelial cell layer), with the hood’s protrusions mainly resulting from marked collagen accumulation in the stratum spongiosum and epithelial cell proliferation, suggesting that the goldfish hood belongs to a cutaneous fibrosis. Furthermore, we found that the down-regulation of arachidonic acid metabolism triggers an inflammatory response, culminating in the dysregulation of the tumor necrosis factor (TNF) pathway, which in turn enhances collagen deposition and epithelial cell proliferation—central to hood morphogenesis. During post-formation process, the aberrant TNF pathway expression and collagen accumulation inhibit osteoclast differentiation, promoting the irregular proliferation of the skull and the formation of bony protrusions that support hood attachment. Our findings not only shed light on the molecular mechanism underlying cutaneous fibrosis in goldfish but also offer potential parallels to analogous conditions in humans.

## Introduction

Goldfish (*Carassius auratus*) is a preeminent ornamental fish originated from polyploid Carassius complex [1–4]. According to the available historical data, the domestication of goldfish began from the Jin Dynasty of China [5]. More than 300 strains with diverse morphological phenotypes have been established during the 1,800 years of domestication process [1,5–8]. “With the most extraordinary modifications by of structure”, goldfish was fascinated by Charles Darwin who gradually developed the theory of “natural selection” [9]. Now, it is considered as an excellent model organism for genetics, evo-devo biology, and comparative endocrinology studies [10–16].

Recently, the mysteries underlying goldfish morphological variations are beginning to unravel along with the genome sequencing and multi-omics analysis [10,17–19]. RNA sequencing revealed that up-regulation of “PPAR (peroxisome proliferator-activated receptor) signaling pathway”, accompanied by an increase in lipid accumulation, might be related to morphological and structural transformation of dragon-eye goldfish [17]. Genome-wide association studies revealed 6 candidate genetic loci and 2 genomic regions that might be associated with several phenotypes, including twin-tail, long-tail, heart-shaped tail, dorsal fin loss, telescope-eye (dragon-eye), albino, and transparent scale [18,19]; only *low-density lipoprotein receptor-related protein 2B* (*lrp2aB*), *oculocutaneous albinism type II (oca2)*, and *chordin* (*chdA*) have been verified as causal genes for the dragon-eye, albino, and twin-tail by using CRISPR/Cas9-mediated gene editing [10,20]. However, the underlying genetic pathways and formation mechanisms of other morphological variations are still largely unknown.

Warty growth or hood is a unique ornamental phenotype in goldfish charactered by thickened epidermis of cranial and opercula regions. Several strains, such as Lionhead and Ranchu, show remarkable warty growth around the cranium at the late developmental stages [21]. Although this mutated trait was described 100 years ago in goldfish [22], the molecular basis underlying the developmental process is still totally unknown. Only a few studies revealed the histological structure and ultrastructure of hoods [23–25]. By using suppression subtractive hybridization, several genes involved in connective tissue and tumor growth were screened to show differential expression between hoods (1 year old) and normal epithelial tissue (40 d after hatching) on head of Red-Lionhead goldfish [26]. However, whether hoods are benign tumor or not is controversial [25]. Therefore, the formation mechanism of hoods has not been clarified. In this study, we first characterized the goldfish hood as a cutaneous fibrosis and documented the irregular proliferation and skull protrusions in Lionhead goldfish. Then, we uncovered the dynamic changes in pivotal pathways throughout the hood’s morphogenesis and progression. These findings not only elucidate the molecular mechanisms underlying cutaneous fibrosis in goldfish with hoods but also provide insights that may be relevant to analogous human conditions.

## Results

### Structure and collagen-dominant composition of the hoods of Lionhead goldfish

**Fig. 1.**
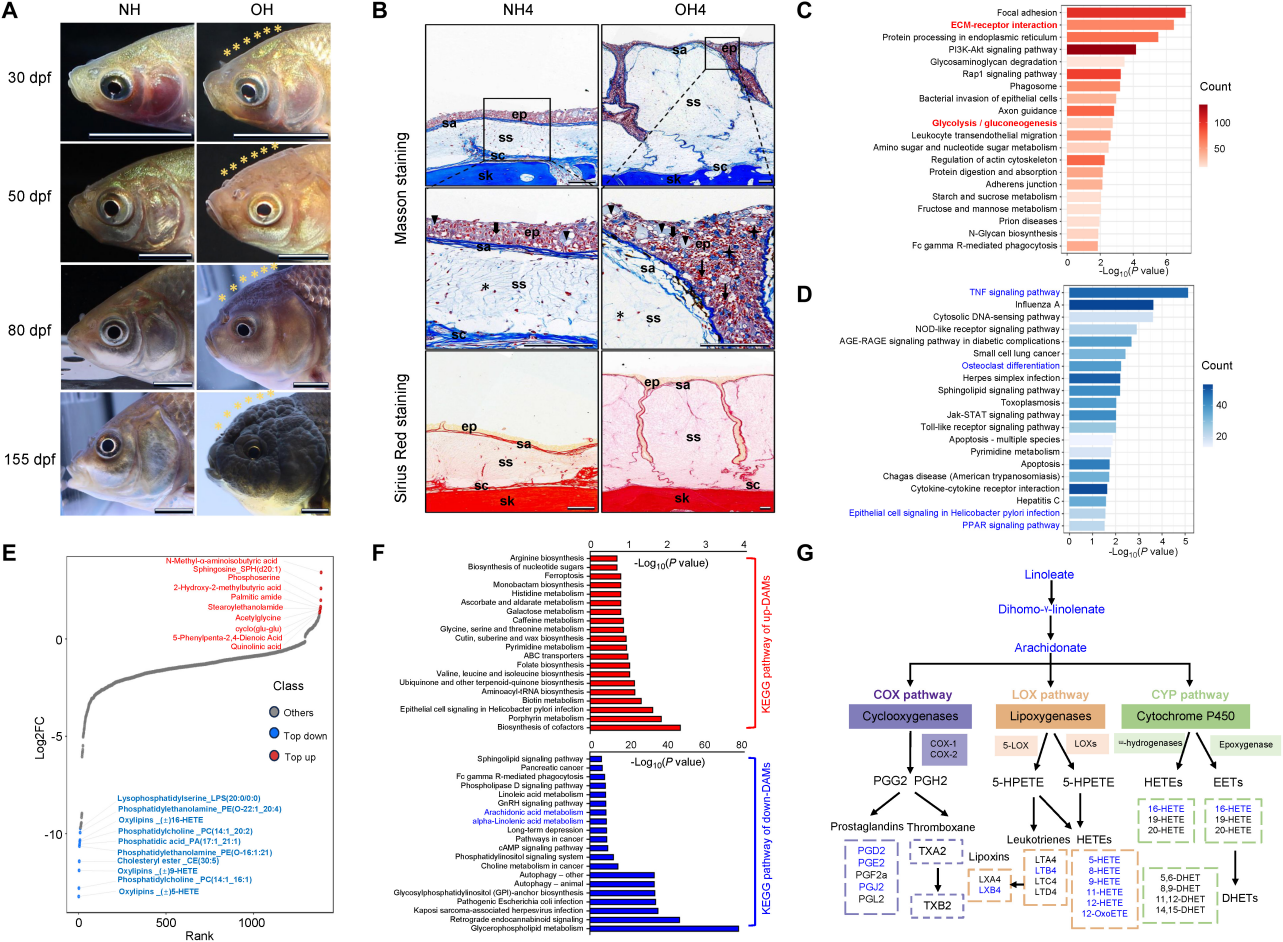
Comparative morphological and multi-omics analysis of the head skin between WT crucian carp with normal hood (NH) and Lionhead goldfish with Oranda Hood (OH). (A) Morphogenetic processes of NH and OH. (B) Histological structure and fibrosis degree of head skin sections assessed by Masson’s trichrome and Sirius Red staining, with blue in Masson and red in Sirius Red staining indicating positive staining (ep, epidermis; sa, stratum adventitia; ss, stratum spongiosum; sc, stratum compactum; sk, skull; triangle, mucous cell; fine arrow, inflammatory cell; thick arrow, epithelial cell; 4-pointed star, cells with collagen deposition; hexagram, fibroblasts). (C) Top 20 KEGG pathways enriched by up-regulated DEUs in OH4 versus NH4. The *x* axis denotes the enrichment factor for each pathway, and the *y* axis labels the pathways. Color intensity corresponds to the number of DEUs associated with each pathway. (D) Top 20 KEGG pathways enriched by down-regulated DEUs in OH4 versus NH4. Axis descriptions are as in (C). (E) Top 10 differentially abundant metabolites (DAMs) from OH4 versus NH4. The *x* axis represents the cumulative number of metabolites ordered by fold change, and the *y* axis shows the log_2_ fold change. Each point corresponds to a metabolite, with blue points indicating the top 10 down-DAMs and red points indicating the top 10 up-DAMs. (F) Top 20 KEGG pathways enriched by up-DAMs (red) and down-DAMs (blue) from OH4 versus NH4. Axis descriptions are as in (C). (G) Schematic of the linoleic acid to AA metabolic pathway, with blue font highlighting down-DAMs.

The hoods of Lionhead goldfish begin to develop approximately 50 d post-fertilization (dpf) and become distinctly visible at 80 dpf. At 155 dpf, the epidermis has thickened to encompass the entire cephalic region, including both the cranial and opercular areas (Fig. 1A). To delineate the histological structure and principal components, we employed Masson’s trichrome and Sirius Red staining on sections of the head skin, both of which are proficient in accentuating collagen fibers within tissue contexts. The epidermis overlying the skull in both wild-type (WT) crucian carp and Lionhead goldfish exhibits a stratified architecture consisting of 4 distinct layers: From the basal to the superficial layer, these are designated as stratum compactum, stratum spongiosum, stratum adventitia, and epithelial cell layer, respectively. In contrast to the WT crucian carp, Lionhead goldfish exhibit a significant hyperplasia of the stratum spongiosum, which is distinguished by a profusion of loosely arranged collagen fibers that are distinctly rendered light blue by Masson’s trichrome staining (Fig. 1B). The stratum adventitia and stratum compactum are delineated by a dense, interwoven network of collagen fibers. The Lionhead goldfish epidermis exhibits pronounced invaginations, with an observable increase in the number of cells staining intensely blue, suggesting a significant deposition of collagen. This pattern is consistent with the results obtained from Sirius Red staining. Collectively, these observations suggest that the hoods of Lionhead goldfish are characterized by an abundance of fibrillar collagen. Furthermore, an augmentation of mucous cells and infiltrating inflammatory cells within the hoods implies the presence of immune dysregulation (Fig. 1B).

### Fatty acid metabolism dysregulation in fibrotic hoods

To uncover the gene expression patterns and metabolic shifts underlying the development of excrescences in Lionhead goldfish, we conducted an integrated transcriptomic and metabolomic analysis. Samples were collected from the hoods of Lionhead goldfish at 155 dpf (OH4) and from the normal head skin of age- and site-matched WT crucian carp (NH4). Principal components analysis (PCA) revealed a pronounced divergence between the 2 groups (Fig. S1A). We identified a total of 10,412 differentially expressed unigenes (DEUs), comprising 4,951 up-regulated and 5,461 down-regulated DEUs (Fig. S1B and Table S2). Kyoto Encyclopedia of Genes and Genomes (KEGG) analysis indicated that the most significant pathways enriched by up-regulated DEUs in the hoods included “ECM-receptor interaction” and “glycolysis/gluconeogenesis” and pathways implicated in immune and metabolic processes (Fig. 1C). The extracellular matrix (ECM), which is composed of macromolecules such as collagen and fibronectin, exhibits an up-regulation of this pathway that aligns with the collagen deposition observed in the stratum spongiosum of the hoods. Conversely, the majority of the top 20 down-regulated pathways were linked to immune responses and disease, such as the “TNF (tumor necrosis factor) signaling pathway” and “epithelial cell signaling in *Helicobacter pylori* infection” (Fig. 1D). Additionally, the lipid metabolism-related “PPAR signaling pathway”, as well as the bone formation-related “TNF signaling pathway” and “osteoclast differentiation” were also enriched.

Furthermore, we identified 1,386 differentially accumulated metabolites (DAMs; 92 up-regulated and 1,294 down-regulated) from OH4 versus NH4 (Fig. S1D). Among these DAMs, *N*-methyl-a-aminoisobutyric acid was uniquely identified, exhibiting a significant over 10-fold increase in concentration within the hoods. The up-regulated DAMs were notably enriched in pathways such as “glycine, serine and threonine metabolism” and “galactose metabolism”. Strikingly, the 10 most DAMs, including the oxylipins 5-HETE, 9-HETE, and 16-HETE, exhibited substantial down-regulation in the hoods, ranging from 850- to 9,500-fold (Fig. 1E and Table S3). KEGG pathway analysis indicated that these down-regulated DAMs were significantly enriched in pathways associated with “glycerophospholipid metabolism”, “alpha-linolenic acid metabolism”, “arachidonic acid metabolism”, and “linoleic acid metabolism” (Fig. 1F). Collectively, these findings suggest a potential dysregulation of fatty acid metabolism. Significantly, linoleic acid, a crucial precursor for the synthesis of arachidonic acid (AA) [27,28], along with its metabolite, such as 5-HETE, 9-HETE, and 16-HETE, was found to be diminished in the hoods (Fig. 1G), indicating a possible disruption in the AA metabolic pathway.

### Inflammatory response is triggered in hood development

**Fig. 2.**
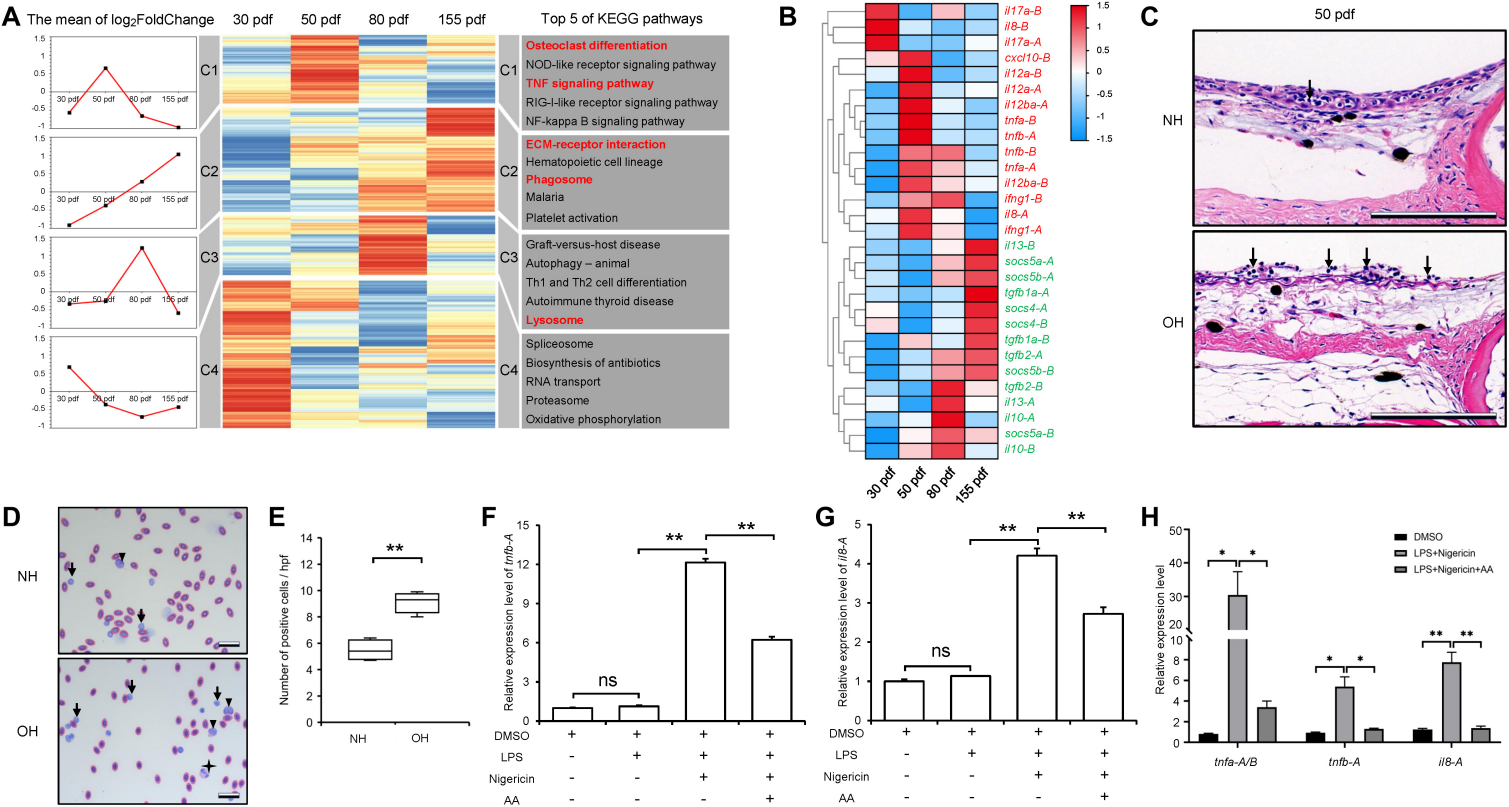
Expression pattern clustering and AA-mediated inflammatory responses during the morphogenesis and development of the hood. (A) Cluster analysis of DEUs across 4 developmental stages of hood and top 5 enriched pathways for each cluster. (B) Heatmap of inflammatory factor gene expression. Red font indicates pro-inflammatory factors, and green indicates anti-inflammatory factors. Color intensity corresponds to the log_2_ value of the fold change. (C) Histological structure of head skin sections from WT crucian carp and Lionhead goldfish stained with H&E. Arrow indicates an inflammatory cell. (D) Giemsa staining of peripheral blood cells from WT crucian carp and Lionhead goldfish. (E) Statistical analysis of inflammatory cell counts in (D). Arrows, triangles, and 4-pointed stars represent lymphocytes, macrophages, and granulocytes, respectively. *n* = 4. (F) AA inhibits the expression of pro-inflammatory factors *tnfb-A* (F) and *il8-A* (G) induced by LPS and nigericin (*n* = 3). (H) Relative expression levels of *tnfa-A/B*, *tnfb-A*, and *il8-A* in hood tissues of live Lionhead goldfish after AA injection versus vehicle controls. DMSO was used as a control. To activate inflammation, LPS (20 mg/kg) and nigericin (4 mg/kg) were co-injected. The experimental group received co-injections of LPS, nigericin, and AA (150 mg/kg). *n* = 4. Data represent mean ± SEM; *P < 0.05, **P < 0.01 (Student’s *t* test).

To further elucidate the gene expression characteristics during the hood’s morphogenesis and progression, we comparatively analyzed the head skin transcriptomes of Lionhead goldfish and WT crucian carp at 4 developmental stages: 30 dpf (OH1 versus NH1), 50 dpf (OH2 versus NH2), 80 dpf (OH3 versus NH3), and 155 dpf (OH4 versus NH4) (Fig. 1A). A union of DEUs called from the 4 comparative transcriptomes included 26,177 DEUs (Fig. S1B and Table S2). Hierarchical clustering analysis showed they were categorized into 4 clusters, displaying different temporal expression patterns (clusters C1 to C4) (Fig. 2A). The DEUs within C1 (7,175 DEUs) exhibited a consistent up-regulation across all 4 developmental stages. Pathway analysis of C1 revealed an early and sustained increase in “ECM-receptor interaction”, consistent with collagen-dominant characteristics of goldfish hoods. The DEUs in cluster C2 containing 4,707 up-regulated genes only at 50 dpf were mainly enriched to “TNF signaling pathway”, “osteoclast differentiation”, “NOD-like receptor signaling pathway”, and other immune and inflammatory pathways, implying that hoods are under inflammatory state at the onset of hood growth. Cluster 3 comprised 4,085 DEUs specifically up-regulated at 80 dpf, which were significantly enriched in pathways related to “autophagy” and “lysosome” and those implicated in autoimmune or immune rejection processes. In C4, the top 10 enriched pathways for the DEUs encompassed “spliceosome”, “RNA transport”, “proteasome”, and others.

Next, we investigated whether the inflammatory response is triggered in the goldfish hood growth. A selection of key pro- or anti-inflammatory factors were subjected to hierarchical clustering analysis [29,30] (Fig. 2B). In comparison with head skin of WT crucian carp, several pro-inflammatory cytokines, such as 2 homeologs of interleukin-17 (*il17-A* and *il17-B*) and *il8-B* (homeolog of *il8* in B subgenome of goldfish), were up-regulated in hoods at 30 dpf. Subsequently, the expression of other pro-inflammatory factors, including *tnfα* (*tumor necrosis factor alpha*), *il12*, *ifng1* (*interferon gamma 1*), and *cxcl10* (*C-X-C motif chemokine 10*), was up-regulated at 50 dpf. As the hoods of goldfish developed between 80 and 155 dpf, there was a significant shift from a pro-inflammatory to an anti-inflammatory state. This transition was marked by the repression of pro-inflammatory genes and the activation of anti-inflammatory genes; the latter included *tgfb1* (*transforming growth factor-β1*), *tgfb2* (*transforming growth factor-β2*), *il-10* (*interleukin10*), *il-13* (*interleukin 13*), *socs4* (*suppressor of cytokine signaling 4*), and *socs5* (*suppressor of cytokine signaling 5*). These dynamic expression changes underscore the ongoing inflammatory processes that occurred during hood morphogenesis and progression. To confirm this transcriptional signature detected in hoods, we examined the histological structure of head skin and the proportion of peripheral blood cells. A significantly higher presence of inflammatory cells, including lymphocytes and macrophages, was observed in the epidermis of hoods and peripheral blood of Lionhead goldfish compared to WT crucian carp at 50 dpf (Fig. 2C to E). Thus, the data confirm the initiation of an inflammatory response in Lionhead goldfish during hood development. Significantly, our cellular experiments have demonstrated that a reduction in AA levels led to the activation of inflammatory cytokine gene expression, such as *tnfb*-A and *il8*-A (Fig. 2F and G). In addition, the injection of AA into hood tissues of live Lionhead goldfish similarly suppressed inflammation-related gene expression (Fig. 2H). The above results suggested that the dysregulation of AA metabolism may be a critical factor in inducing inflammatory responses.

### Predominance of fibril-related collagens in the hoods of Lionhead goldfish

**Fig. 3.**
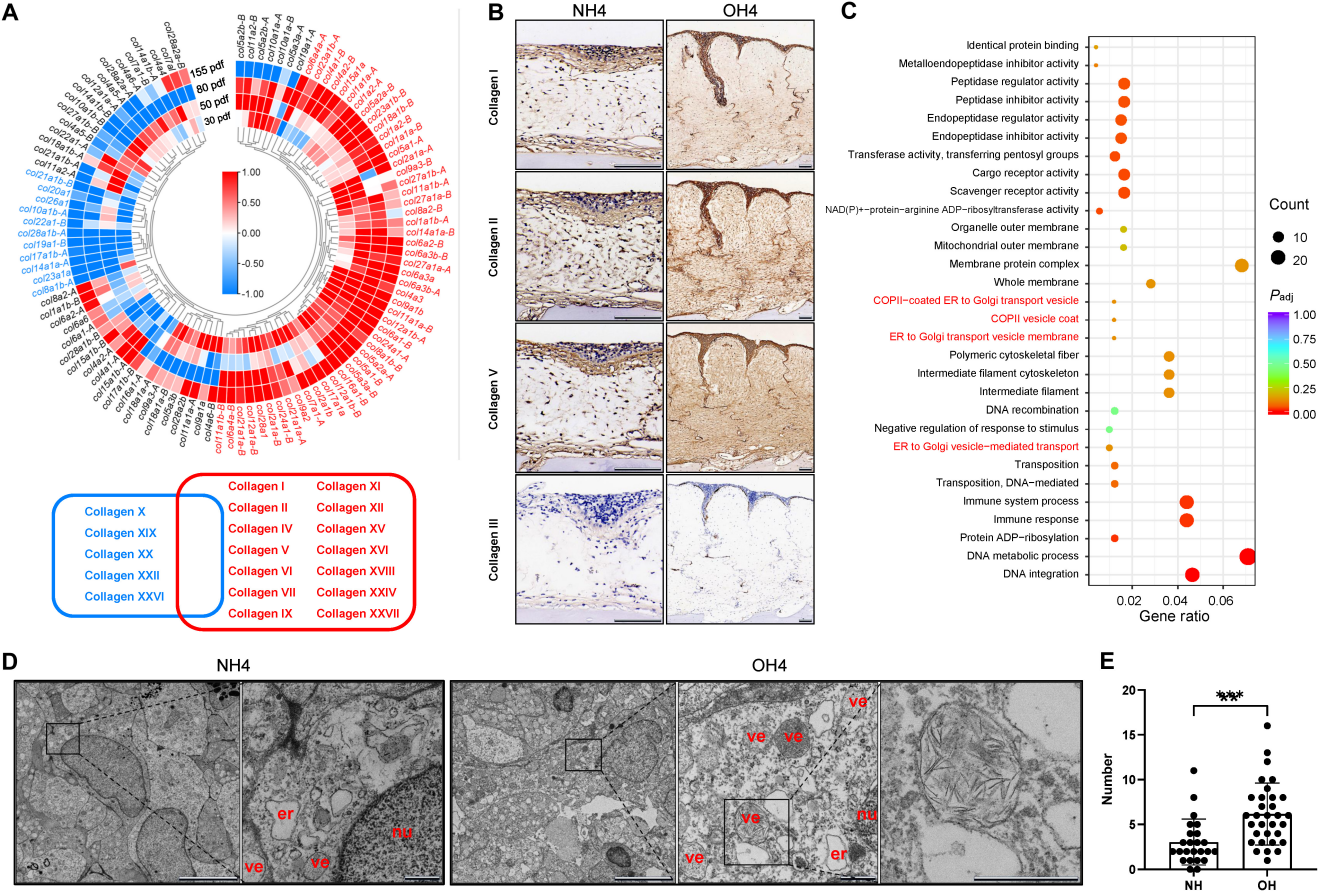
Collagen deposition and the related gene expression in goldfish hood. (A) Expression heatmap of collagen-related genes and key collagen types associated with up-regulated and down-regulated genes. Color intensity corresponds to the log_2_ value of the fold change. (B) Immunohistochemical detection of 4 collagen-related proteins in head skin tissue, with brown indicating positive staining. (C) GO enrichment analysis based on the intersection of DEUs from 4 stages. Red font highlights collagen-related GO terms. The *x* axis represents the gene ratio, and the *y* axis lists the GO terms. (D) Electron micrographs of the stratum spongiosum from WT crucian carp and Lionhead goldfish at 155 dpf. er, endoplasmic reticulum; ve, vesicle; nu, nucleus. Vesicles contain a large amount of fibrous material. (E) Number of vesicles in the spongiosum layer of WT crucian carp and Lionhead goldfish. Data represent mean ± SEM; *n* for NH = 3, *n* for OH = 4; *P < 0.05, **P < 0.01 (Student’s *t* test).

Histological examination of the hoods using both Masson’s trichrome and Sirius Red staining revealed an abundance of loose collagen fibers (Fig. 1B). To elucidate the collagen composition within the hoods, we initially identified all collagen-encoding genes from the transcriptome data and conducted hierarchical clustering analysis (Fig. 3A). During the growth of hoods, the expression of 14 collagen types, which are predominantly involved in the formation of collagen fibers, was found to be consistently up-regulated across all developmental stages. These included fibril-forming collagens (types I, II, V, XI, XXIV, and XXVII) and fibril-associated collagens (types IX, XII, and XVI) [31,32], thereby reaffirming the preeminence of collagen fibers in the hood’s composition. In contrast, the expression of 5 other collagen types, comprising 3 fibril-associated collagens (types XIX, XX, and XXII), one network-forming collagen (type X), and collagen XXVI, remained down-regulated throughout these stages.

Subsequently, to validate the collagen composition within the hoods, we conducted immunohistochemical analyses using antibodies specific for type I, II, III, and V collagens. Our results revealed a significantly higher abundance of type I, II, and V collagens in the hoods compared to the head skin of WT crucian carp (Fig. 3B). In alignment with the transcriptome data, type III collagen was detected only in minimal amounts.

Additionally, we integrated the DEUs common across the 4 developmental stages of OH versus NH comparisons, resulting in an intersection of 1,900 DEUs (Fig. S1C and Table S2). Gene Ontology (GO) enrichment analysis of these shared DEUs revealed a significant association with vesicle-related terms, including “COPII-coated ER to Golgi transport vesicle”, “COPII vesicle coat”, “ER to Golgi transport vesicle membrane”, and “ER to Golgi vesicle-mediated transport”, with the term “COPII vesicle coat” being particularly enriched (Fig. 3C, Fig. S2, and Table S4). Consequently, we examined the ultrastructure of the hoods using transmission electron microscopy (TEM). A markedly higher number of spherical vesicles were observed in the stratum spongiosum of the hoods compared to the WT crucian carp; some of these vesicles appeared to contain irregular filaments (Fig. 3D and E). Collectively, these findings confirm an enhanced deposition of collagen fibers within the hoods of Lionhead goldfish.

### TNF-mediated collagen deposition and subsequent epithelial cell proliferation

**Fig. 4.**
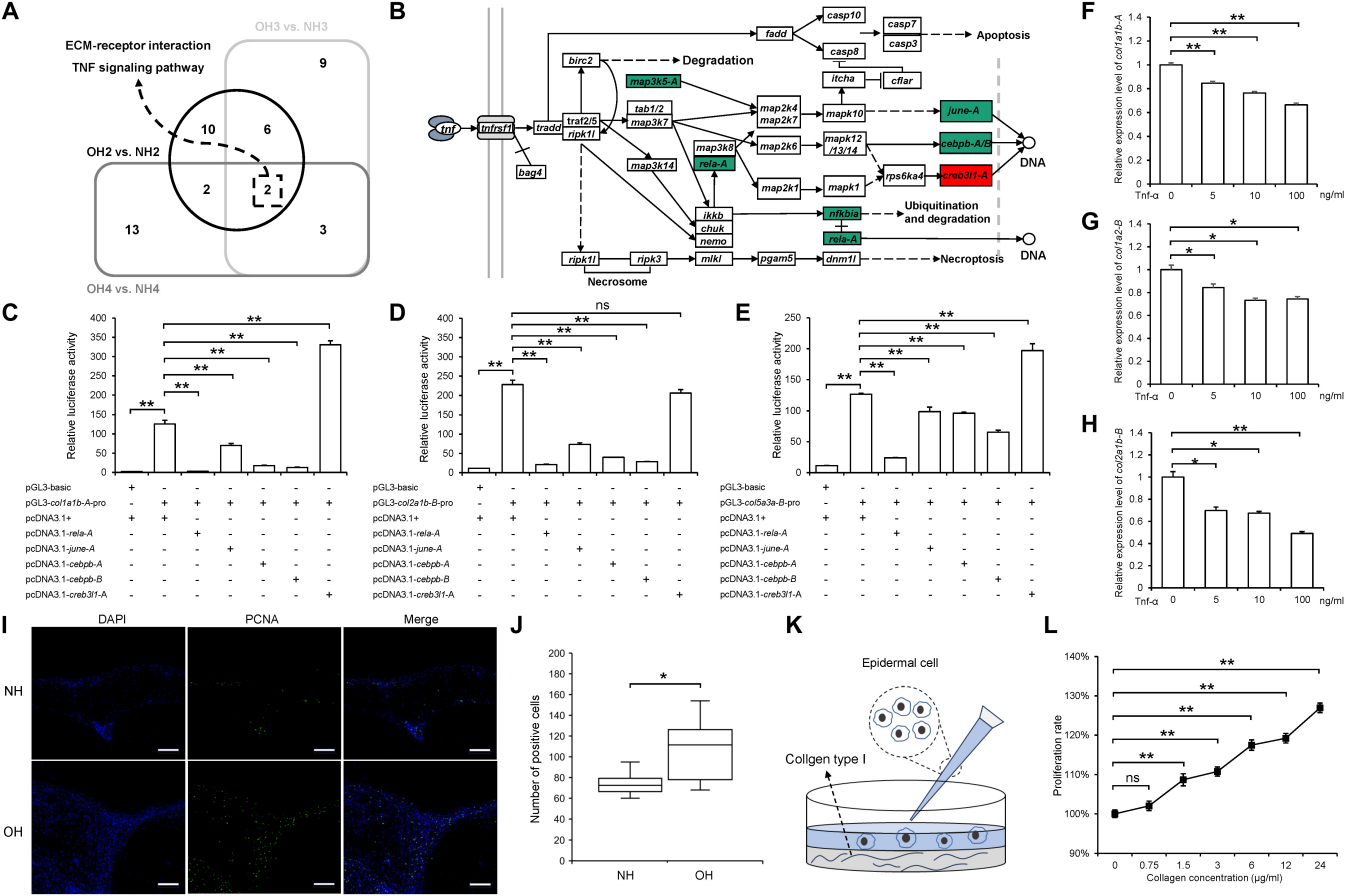
Down-regulation of the TNF signaling pathway promotes collagen deposition in hood and thereby enhances epithelial cell proliferation. (A) Venn diagram of the top 20 enriched pathways during the 3 developmental stages of hood (OH2 versus NH2, OH3 versus NH3, and OH4 versus NH4), with 2 common pathways in the intersection. (B) TNF signaling pathway, with red indicating genes up-regulated in all 4 stages, green indicating genes down-regulated in all 4 stages, and white indicating genes that are not differentially expressed or are not uniformly up-regulated or down-regulated across all 4 stages. (C to E) Dual-luciferase assays of transcription factors *rela-A*, *june-A*, *cebpb-A*, *cebpb-B*, and *creb3l1-A* on the promoter activity of *col1a1b-A* (C), *col2a1b-B* (D), and *col5a3a-B* (E). *n* = 3. (F to H) Expression changes of collagen-related genes *col1a1b-A* (F), *col1a2-B* (G), and *col2a1b-B* (H) after treatment of primary fibroblasts derived from Lionhead goldfish hoods with recombinant TNF-α protein, *n* = 3. (I) Immunofluorescence staining of WT crucian carp and Lionhead goldfish hood sections at 155 dpf using an anti-PCNA antibody. (J) Quantification of PCNA-positive cell counts in the high-power field of WT crucian carp and Lionhead goldfish (*n* = 8). (K) Schematic representation of the experiment investigating the effect of collagen content on epithelial cell proliferation. (L) Proliferation rate of epithelial cells after treatment with different concentrations of type I collagen. *n* = 10. Data represent mean ± SEM; *P < 0.05, **P < 0.01 (Student’s *t* test).

We identified a convergence on 2 significant pathways, “ECM-receptor interaction” and the “TNF signaling pathway”, by intersecting the top 20 enriched pathways from DEUs associated with the hood morphogenesis and progression (OH2 versus NH2, OH3 versus NH3, and OH4 versus NH4) (Fig. 4A and Fig. S3). Moreover, DEUs from clusters C1 and C2, as determined by hierarchical clustering analysis, were notably enriched in these pathways, suggesting their crucial roles in the morphogenesis and progression of hoods (Fig. 2A). Given the established effects of inflammation on the excessive deposition of a collagen-rich ECM and the pivotal role of the “TNF signaling pathway” in inflammation [33,34], we investigated whether the down-regulation of the “TNF signaling pathway” after 80 dpf is implicated in hood formation. Within the “TNF signaling pathway” (Fig. 4B and Fig. S4), 5 transcription factors were identified, with 4—*rela-A* (*NF-kappa-B transcription factor p65-like*), *junE-A* (*transcription factor AP-1-like*), *cebpb-A*, and *cebpb-B* (*CCAAT/enhancer-binding protein beta-like*)—showing a down-regulated expression trend, while only *creb3l1-A* (*cyclic AMP-responsive element-binding protein 3-like protein 1*) was consistently up-regulated across the 4 periods. To ascertain the influence of the “TNF signaling pathway” on collagen synthesis, we conducted dual-luciferase reporter assays. In *C. auratus* blastulae (CAB) cells, the overexpression of *rela-A*, *june-A*, *cebpb-A*, and *cebpb-B* significantly inhibited the promoter activities of collagen indicator genes, such as *col1a1b-A* (*collagen, type I, alpha 1b*), *col2a1b-B* (*collagen, type II, alpha 1b*), and *col5a3a-B* (*collagen, type V, alpha 3a*), whereas the overexpression of *creb3l1-A* enhanced the promoter activities of *col1a1b-A* and *col5a3a-B* (Fig. 4C and D). These results imply that the down-regulated expression of *rela-A, june-A*, *cebpb-A*, and *cebpb-B*, in conjunction with the up-regulated expression of *creb3l1-A*, may be instrumental in the excessive collagen deposition in goldfish hoods. Furthermore, goldfish recombinant protein TNFα induced a dose-dependent decrease in the expression levels of *col1a1b-A*, *col2a1b-B*, and *col1a2-B* in primary fibroblasts derived from Lionhead goldfish hoods (Fig. 4F to H). This indicates that the down-regulation of *tnfα* expression after 80 dpf may be associated with the excessive collagen deposition in goldfish hoods.

Considering the hyperplastic phenotype of the epidermis in the hoods (Fig. 1B), we performed an immunofluorescence assay for proliferating cell nuclear antigen (PCNA) to assess the difference in cell proliferation between the hoods and the normal head skin of WT crucian carp. As illustrated in Fig. 4I and J, the number of PCNA-positive cells in the hoods was significantly higher than that in WT crucian carp. Moreover, we utilized a cell counting kit-8 (CCK-8) assay to evaluate the cell proliferation of hood primary epithelial cells cultured on plates coated with type I collagen (Fig. 4K). The results demonstrated a pronounced dose-dependent proliferation in response to collagen exposure (Fig. 4L).

### Dysregulation of osteoclast differentiation initiates abnormal skull morphogenesis in hood development

**Fig. 5.**
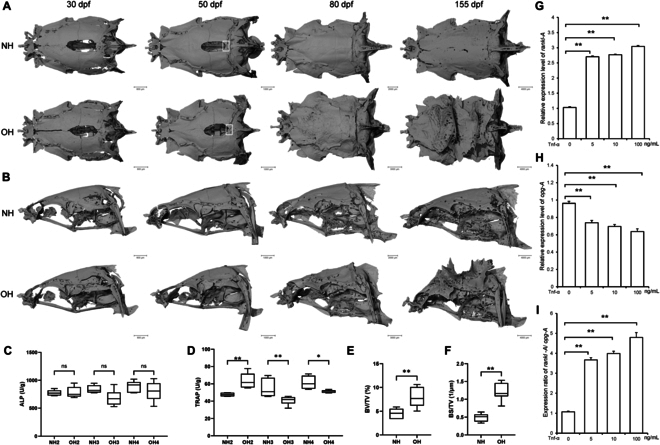
The development of the hood is accompanied by the emergence of irregular skull protrusions. (A) Dorsal and (B) lateral views of micro-CT scans and 3-dimensional reconstructions of skulls from WT crucian carp and Lionhead goldfish. (C and D) Activity of alkaline phosphatase (C) and tartrate-resistant acid phosphatase (D) in the skull across the 3 developmental stages of the hood. *n* = 6. (E and F) Bone volume to total volume (BV/TV) (E) and bone surface to total volume (BS/TV) (F) ratios in the skulls of WT crucian carp and Lionhead goldfish at 155 dpf. *n* = 7. Relative expression levels of *rankl-A* (G) and *opg-A* (H) in fibroblasts from goldfish hoods. *n* = 3. (I) *rankl-A*/*opg-A* ratio following treatment with recombinant TNF-α protein. *n* = 3. Data represent mean ± SEM; *P < 0.05, **P < 0.01 (Student’s *t* test).

Intriguingly, the pathway most significantly enriched by the DEUs from cluster C2, as determined by hierarchical clustering analysis, was “osteoclast differentiation” (Fig. 2A). This finding prompted us to examine skull morphology using micro-computed tomography (micro-CT) (Fig. 5A and B) and to evaluate osteoclastic and osteoblastic activities, as indicated by alkaline phosphatase (ALP) (Fig. 5C) and tartrate-resistant acid phosphatase (TRAP) (Fig. 5D) activities, respectively. During the morphogenetic stage of hood, ranging from 30 to 50 dpf, there was an up-regulation of osteoclast differentiation. This was concurrent with a disruption in the osseous fusion of the cranial parietal bones, leading to a failure in the proper healing of the parietal bones of the skull (Fig. 5A). Conversely, as the hood tissue progressively develops and accumulates, the osteoclast differentiation pathway is down-regulated, coinciding with the abnormal hyperplasia of the skull. These skeletal changes were accompanied by a significant decrease in TRAP activities (Fig. 5D). By 155 dpf, the irregular protuberances on the goldfish skull had become increasingly pronounced, with significantly higher BV/TV (bone volume/tissue volume, percent bone volume) and BS/TV (bone surface/tissue volume, bone surface density values) compared to the WT crucian carp (Fig. 5E and F), indicating that bone anabolism exceeds catabolism, leading to increased bone mass in Lionhead goldfish. In contrast, ALP activities in the skull of Lionhead goldfish did not significantly differ from those of the WT crucian carp, suggesting similar levels of osteoblastic differentiation (Fig. 5C). Conversely, the TRAP activities indicated that osteoclast differentiation in the skull of Lionhead goldfish was reduced compared to the WT crucian carp at 80 and 155 dpf (Fig. 5D), suggesting that the skull protuberances may be attributed to decreased osteoclast differentiation.

To elucidate the cause of decreased osteoclast differentiation, we considered the TNF signaling pathway, which was enriched throughout the 4 stages of hood development and is known to induce osteoclast differentiation [35–38]. We stimulated primary cells to assess the relative expression levels of *rankl-A* (*receptor activator of nuclear factor-kappa B ligand*), a key promoter of osteoclast differentiation and bone resorption, and *opg-A*, a soluble member of the TNF superfamily that positively regulates bone density [39,40]. Our results showed that treatment with recombinant protein TNF-α enhanced the expression of *rankl-A* mRNA (Fig. 5G), suppressed the expression of *opg-A* mRNA (Fig. 5H), and increased the *rankl-A*/*opg-A* ratio (Fig. 5I).

**Fig. 6.**
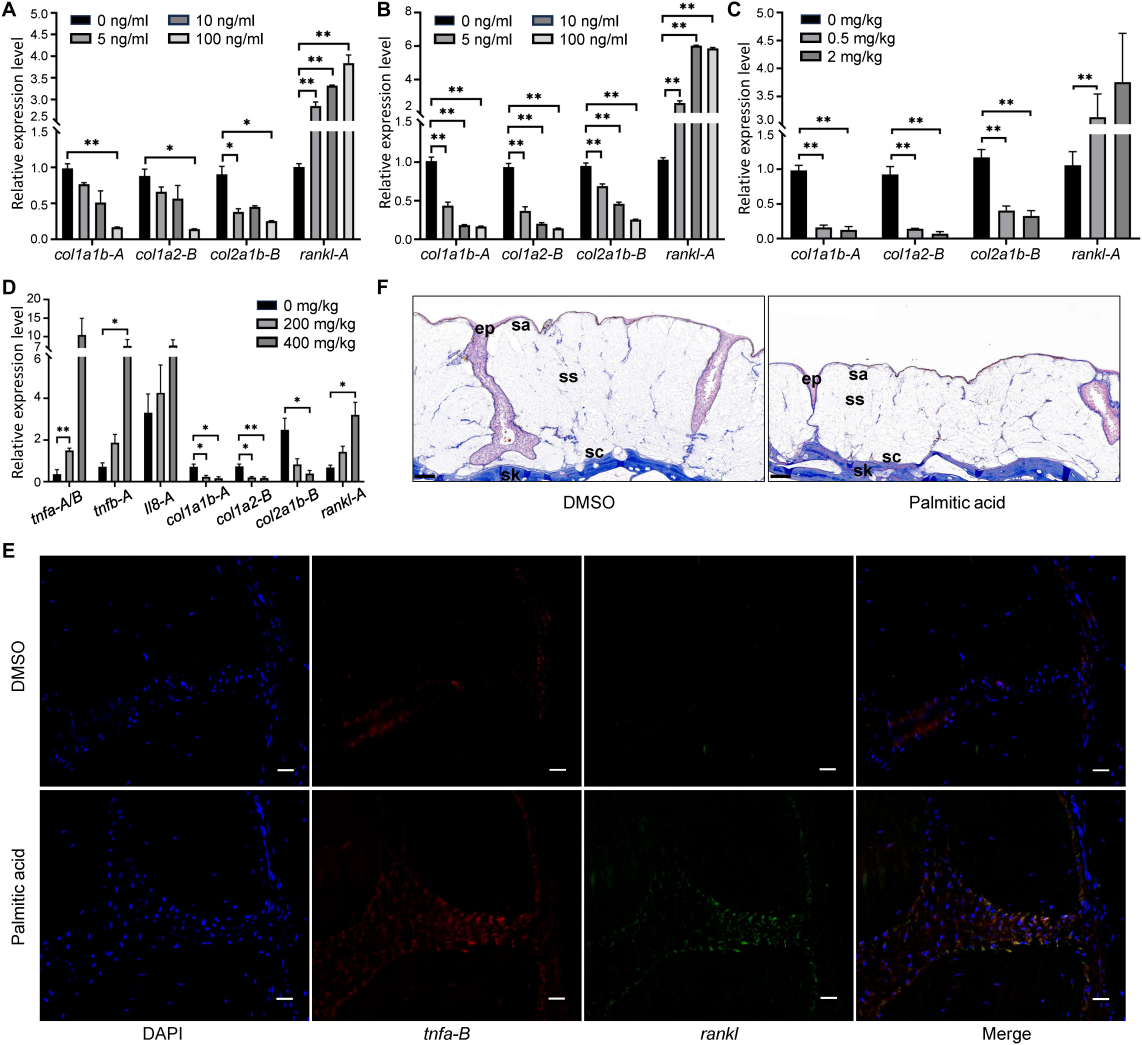
Functional validation of the regulation among TNF pathway, collagen deposition, and osteoclast differentiation in vivo and in vitro*.* Relative expression levels of *col1a1b-A*, *col1a2-B*, *col2a1b-B,* and *rankl* in TNF-α-treated GICS cells (A) and ex vivo hood tissue explants (B). *n* = 3. (C) Relative expression levels of collagen genes and *rankl-A* in hood tissues after recombinant TNF-α protein injection. *n* = 4. (D) Relative expression levels of *tnfa-A/B*, *tnfb-A*, *il8-A*, *col1a1b-A*, *col1a2-B*, *col2a1b-B*, and *rankl-A* in hood tissues of live Lionhead goldfish after PA injection versus vehicle controls. *n* = 4. (E) RNA-FISH of *tnfa-B* (red) and *rankl-A* (green) in the skull of hood tissues injected with PA and DMSO controls. Blue fluorescence is stained by DAPI. Scale bar, 20 μm. (F) Histological analysis of PA-treated hoods (versus DMSO control) using Masson staining, with blue indicating positive staining. Scale bar, 200 μm. Data represent mean ± SEM; *P < 0.05, **P < 0.01 (Student’s *t* test).

Moreover, we detected the down-regulated expression of collagen gene (*col1a1b-A*, *col1a2-B*, and *col2a1b-B*) and the up-regulated expression of *rankl-A* in gibel carp skin (GICS) cells (Fig. 6A) and ex vivo hood tissue explants (Fig. 6B) treated with recombinant TNF-α protein. We also injected TNF-α protein into the hoods of live Lionhead goldfish and observed similar results (Fig. 6C). Collectively, these findings demonstrate that the down-regulation of TNF signaling during hood expansion reduced collagen deposition and osteoclast differentiation, thereby leading to the increased bone mass and irregular osseous protrusion formation.

To confirm the regulation among TNF pathway, collagen deposition, and osteoclast differentiation, we conducted rescue experiments by injecting palmitic acid (PA), which can induce a pro-inflammatory microenvironment [41], into hood tissues at 80 dpf to reactivate TNF signaling. PA treatment up-regulated the expression of *tnfa-A/B*, *tnfb-A*, and *il8-A*. This reactivation of TNF signaling reduced the expression of *col1a1b-A*, *col1a2-B*, and *col2a1b-B* and enhanced the expression of *rankl-A* (Fig. 6D). After 40 d of consecutive injections, we observed up-regulated *tnfa-B* and *rankl-A* in the skull of the hood, with their expressions colocalizing, using RNA fluorescence in situ hybridization (FISH) (Fig. 6E). Additionally, the stratum spongiosum dominated with collagen were thinner compared to controls (Fig. 6F). The above results suggest that TNF signaling pathway affects collagen deposition and osteoclast differentiation.

## Discussion

**Fig. 7.**
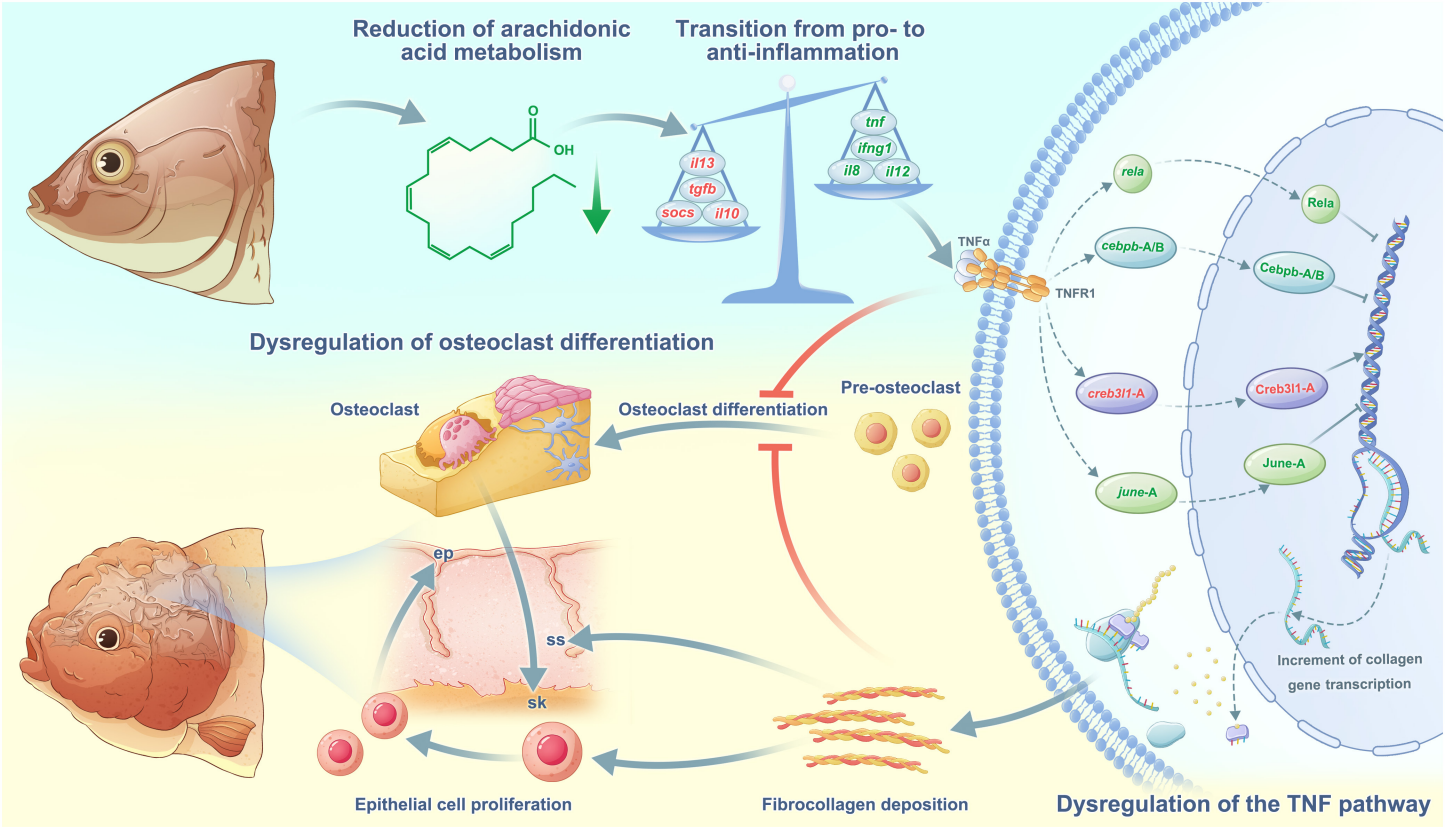
A hypothesized diagram for hood formation in Lionhead goldfish.

The hood, a distinctive cephalic skin protrusion, is a highly prized ornamental feature of goldfish, conferring an adorable and captivating appearance. However, whether hoods are benign tumor or not is controversial, and the etiology of hood formation in goldfish has been poorly understood. Our investigation sheds new light on the hood’s morphogenesis and progression, presenting a comprehensive analysis of the hood’s structural and compositional attributes, along with the transcriptional and metabolic dynamics throughout its development. We discovered that the reduction of AA metabolism triggers an inflammatory cascade, leading to the dysregulation of the TNF pathway. This, in turn, enhances fibrocollagen deposition and stimulates epithelial cell proliferation, which are important drivers of hood morphogenesis. After the formation of hood, the aberrant expression of the TNF pathway and the accumulation of collagen inhibit osteoclast differentiation, promoting bone growth and the development of irregular skull protrusions, which provides an ideal substrate for hood attachment (Fig. 7).

The hood, formed by the protrusion of cephalic skin, has been dissected to reveal a composition rich in mucus and connective tissue [42]. Our histological analysis indicates that goldfish hoods exhibit a 4-layer structure above the skull, with the most significant volumetric expansion attributed to the cell proliferation and thickness of the connective tissue-enriched stratum spongiosum and the epithelial cell layer (Fig. 1A and B). Inflammatory cells were also observed within the hood (Fig. 2D and E). Biochemical analyses by Wang and Chen [43] have shown that the hood’s composition is marked by a high water content exceeding 90%, with proteins constituting approximately 60% and lipids about 30% of the solid content. We indicated that the connective tissue, a complex of cells, fibers, and extracellular matrix, was predominantly collagenous in the stratum spongiosum layer as evidenced through tissue staining and immunohistochemistry (Fig. 3B). “Extracellular matrix”, “extracellular region part”, and “collagen trimer” were enriched by 724 DEUs, which remained up-regulated throughout the 4 periods using GO enrichment analysis (Fig. S1E and F and Table S5). All noncellular components in tissues and organs are collectively referred to as ECM, which not only provides the necessary physical scaffolds for cellular components but also initiates the key biochemical and biomechanical signals needed for tissue morphogenesis, differentiation, and homeostasis [44–46]. The major components of ECMs are fibrous-forming proteins, such as collagens, elastin, fibronectin, laminins, glycoproteins, proteoglycans, and glycosaminoglycans [47–49]. Furthermore, a notable proliferation of fibroblasts was observed within the hood (Fig. 1B), a finding that mirrors the histological alterations typically seen in benign cutaneous fibrous histiocytoma, characterized by elevated levels of collagen and elastic fibers, as well as an increase in fibrohistiocytic cells [50–52]. This observation is congruent with the molecular signature of cutaneous fibrosis, which involves the up-regulation of ECM components and glycolytic pathways, and the down-regulation of PPAR signaling pathway [53,54], a pattern that is corroborated by our data (Fig. 1C and D). In aggregate, these findings suggest that the hood formation in goldfish is attributed to an overabundance of fibrillar collagen deposition, classifying the hood as a benign fibrotic cutaneous neoplasm.

The balance between collagen production and degradation is paramount for the integrity and homeostasis of tissues and organs [55,56]. Collagen gene expression is tightly regulated by a suite of cytokines at the transcriptional level, with transforming growth factor-β (TGF-β) promoting collagen expression and TNF-α exerting an opposing effect [55]. In our study, the TNF signaling pathway was consistently enriched throughout the developmental timeline of goldfish hoods (Figs. 2A and B and 4A), coincident with the down-regulation of 4 transcription factors—*rela-A*, *junE-A*, *cebpb-A*, and *cebpb-B*—and the up-regulation of *creb3l1-A* (Fig. 4A and Fig. S4). Prior research has established that *rela* and *c-jun* can suppress collagen expression [55,57], and that *creb3l1* knockout can diminish collagen production [58]. Our dual-luciferase assays verified that the differential expression of these transcription factors all promotes collagen promoter activity (Fig. 4C to E). Furthermore, the treatment of hood primary fibroblasts, GICS, and ex vivo hood tissue with recombinant TNFα protein demonstrated a dose-dependent inhibitory effect on the expression of collagen-related genes (Figs. 4F to H and 6A and B) and direct injection of recombinant TNFα protein into hood tissues of live Lionhead goldfish similarly down-regulated collagen genes (Fig. 6C). The above results coincide with the down-regulation of the TNF signaling pathway during the rapid growth phase of the hood. These findings underscore the role of TNF signaling dysregulation in promoting collagen accumulation in goldfish hoods. Moreover, given the established correlation between collagen and cell proliferation [59,60], we utilized a CCK-8 assay to evaluate the cell proliferation of hood primary epithelial cells cultured on plates coated with collagen and revealed a pronounced dose-dependent proliferation in response to collagen exposure (Fig. 4K and L). Synthesizing these observations, we conclude that the dysregulation of the TNF signaling pathway triggers the deposition of collagen, which in turn induces epidermal hyperplasia, thereby facilitating the development of the hoods.

TNF, a key biomarker of inflammatory pathways, exhibited up-regulation of pro-inflammatory mediators during the pivotal stages of hood development, with anti-inflammatory factors being up-regulated during the hood’s rapid growth phase (Fig. 2B). We identified an increase in inflammatory cells such as lymphocytes, macrophages, and granulocytes in the scalp and blood of goldfish with hoods (Fig. 2C to E). This indicates that inflammation is a constant accompaniment during hood morphogenesis and expansion. We hypothesize that the initial up-regulation of the TNF signaling pathway (pro-inflammatory) primarily activates the inflammatory response during early hood formation. Conversely, its subsequent down-regulation (anti-inflammatory) promotes collagen deposition, thereby facilitating hood development. To test this hypothesis, we injected PA into hoods during the later developmental stage to reactivate the TNF pathway. This intervention led to up-regulated *tnf* expression, down-regulated collagen genes, and thinner hoods (Fig. 6D and F). Furthermore, direct injection of recombinant TNFα protein into hood tissues yielded consistent results, also suppressing collagen gene expression (Fig. 6C). These results indicate that shifting the microenvironment from anti-inflammatory to pro-inflammatory reduces collagen deposition during hood development. This confirms that the transition from a pro-inflammatory to an anti-inflammatory state, regulated by the TNF signaling pathway, is critical for hood morphogenesis.

Additionally, the dysregulation of AA metabolism, known to modulate inflammatory responses [61], was noted (Fig. 1F and G). The presence of 4 double bonds in AA predisposes it to oxidative metabolism, resulting in a diverse array of bioactive metabolites that are crucial for modulating immune and inflammatory responses [62]. Notably, 3 of the top 10 down-regulated metabolites (5-HETE, 9-HETE, and 16-HETE) in our study are derivatives of AA, signifying a diminished metabolic profile for both AA and its downstream products (Fig. 1F and G). Our quantitative experiments in vivo and in vitro further elucidate that AA possesses an inhibitory effect on the expression of pro-inflammatory cytokines, implying that its reduced availability may precipitate inflammatory processes (Fig. 2F to H). AA inhibits inflammation by reducingnucleotide-binding and oligomerization domain-like receptor (NLR) family pyrin domain-containing 3 (NLRP3) inflammasome activity in human and mouse macrophages [61], and its metabolite prostaglandin E2 (PGE2) down-regulates TNF expression [63,64]. In this study, PGE2 was reduced in OH, compared with NH (Fig. 1G), implying that AA down-regulation may modulate the TNF signaling pathway via PGE2. Collectively, our data propose a model wherein the down-regulation of AA triggers the TNF pathway, augmenting collagen deposition and concurrent inflammation.

At present, there is no report on the variation of goldfish skull. In this study, concurrent with the deposition of collagen in the scalp tissue, we are the first to observe the irregular hyperplastic protrusions of the skull (Fig. 5A and B). The elevated BV/TV ratio indicates a predominance of bone synthesis over resorption (Fig. 5E and F). Given the established link between the TNF signaling pathway and osteoclast differentiation [35–38], we stimulated primary cells, GICS cells, and ex vivo hood tissue explants with recombinant TNFα protein, as well as direct injection of recombinant TNFα protein into hood tissues of live Lionhead goldfish. These interventions consistently increased the expression of *rankl*, confirming TNFα’s role in enhancing key molecular markers of osteoclast differentiation (Figs. 5G to I and 6A to C). Moreover, we have confirmed the TNF–osteoclast axis through FISH and quantitative polymerase chain reaction (qPCR) (Fig. 6D and E). These consequences imply that the down-regulation of the TNF pathway in the progression stage of hood could lead to reduced osteoclast differentiation, contributing to the hyperplasia of the goldfish skull. Furthermore, the increased levels of type II and type IX collagen in the scalp tissue of goldfish (Fig. 3A and B), known to inhibit osteoclast formation [65], suggest that collagen deposition might also play a role in skull proliferation. Notably, the up-regulation of *creb3l1*, a transcription factor in the TNF pathway, in the hood of Lionhead goldfish (Fig. 4B and Fig. S4), is intriguing. *creb3l1*^−/−^ mice exhibit severe bone loss, including reduced type I collagen in the bone matrix and decreased osteoblast activity [66], indicating that *creb3l1* up-regulation might promote bone mass increase.

Together, these findings highlight the significant restructuring of the skull in Lionhead goldfish associated with hood morphogenesis and progression. The TNF pathway down-regulation as the hood expands leads to collagen deposition and diminished osteoclast differentiation, resulting in increased bone mass and the formation of irregular osseous protrusions. This altered skull architecture, in contrast to the uniformly smooth skull of WT crucian carp, provides a more favorable substrate for the secure attachment and stabilization of the head warty.

Our study has certain limitations. First, our research focused on the Lionhead (Oranda) goldfish strain. Given the diverse hood morphologies across goldfish strains, including Oranda, Tiger-head, Goose-head, and Crown Pearl-scale, it remains unclear whether the identified molecular mechanisms, particularly the regulation among TNF pathway, collagen deposition, and osteoclast differentiation and its effect on skull hyperplasia, generalize for all strains. Further research is needed to confirm this. Second, although the hood trait is stably inherited, the specific causal gene(s) for this complex phenotype is still unknown. Future studies combining whole-genome resequencing and functional validation are essential to identify the key genetic factors involved in hood formation. Despite these limitations, our findings shed light on the molecular mechanism underlying cutaneous fibrosis in goldfish and offer potential parallels to analogous conditions in humans.

In conclusion, our morphological analysis of goldfish hoods has revealed a complex structure comprising 4 distinct layers, with the protrusions primarily attributed to substantial collagen deposition in the stratum spongiosum and proliferation of epithelial cells. Through multi-omics and cellular experiments, we have identified the goldfish hood as a form of a cutaneous fibrosis. The down-regulation of AA initiates an inflammatory response that leads to the dysregulation of the TNF pathway, promoting collagen deposition and subsequently inducing epithelial cell proliferation, which are the important drivers of hood morphogenesis. Concurrently, the abnormal expression of the TNF pathway and collagen deposition inhibit osteoclast differentiation, thereby promoting the irregular proliferation of the skull and the formation of bony protrusions that facilitate the attachment of the hood.

## Materials and Methods

### Goldfish hood sample collection

The WT crucian carp (NH) and Lionhead goldfish (OH) were artificially inseminated and reared in fish tanks [10] at the National Aquatic Biological Resource Center (NABRC) in Institute of Hydrobiology, Chinese Academy of Sciences. At 30, 50, 80, and 155 dpf, the NH and OH strains were sampled after deep and overdosed anesthesia with styrylpyridine [10]. All procedures in this study were performed following the ethical requirements of Animal Care and Use Committee of Institute of Hydrobiology, Chinese Academy of Sciences.

### Transcriptome and metabolome sequencing and analysis

RNA extraction and library construction were conducted according to the methods described in our previous publications [10]. Sequencing was carried out on the Illumina HiSeq 4000 platform by Novogene Co. Ltd. Clean reads were aligned to the *C. auratus* genome sequence, as previously determined [67], using HISAT2 v2.0.5. Assembly of new transcripts was performed with StringTie v1.3.3b. Gene expression levels were quantified using fragments per kilobase of exon model per million mapped fragments (FPKM) values, applying a threshold of FPKM > 1, as determined by featureCounts v1.5.0-p3 and StringTie v1.3.3b. DEUs were filtered using a threshold of log_2_(FoldChange)| > 1 and *P*_adj_ ≤ 0.05 employing DESeq2 v1.16.1. These DEUs were then utilized for subsequent GO and KEGG enrichment analyses, conducted with clusterProfile v3.4.4.

The metabolism was determined by MetWare (http://www.metware.cn/) based on the AB Sciex QTRAP 6500+ LC-MS/MS (liquid chromatography–tandem mass spectrometry) platform. The preparation and extraction were performed according to Zhang et al.’s methods [68]. The sample extracts were analyzed using an LC–electrospray ionization (ESI)–MS/MS system [ultra-performance liquid chromatography (UPLC), ExionLC AD, https://sciex.com.cn/; MS, QTRAP System, https://sciex.com/]. Qualitative metabolite analysis was conducted utilizing retention times, characteristic ion pair information, and secondary spectral data extracted from the target standard database curated by MetWare. Quantitative metabolite analysis was performed using the multiple reaction monitoring (MRM) mode on a triple quadrupole linear ion trap mass spectrometer (QTRAP), specifically the QTRAP LC-MS/MS System. This system, equipped with an ESI turbo ion-spray interface, was operated in both positive and negative ion modes and controlled by Analyst 1.6.3 software. Metabolites that accumulated differentially between groups were identified using the variable importance in the projection (VIP) score, with a threshold of VIP > 1 and a *P* value of <0.05. Subsequently, the differential metabolites were mapped to metabolic pathways based on the KEGG database [69].

### Histological, immunohistochemistry, and immunofluorescence analysis and FISH

Fresh anticoagulated blood samples were uniformly spread onto glass slides and fixed with methanol for 15 min prior to staining with Giemsa (Servicebio, China). Hood samples were postfixed in 4% paraformaldehyde at 4 °C overnight. Following dehydration and embedding, sections of 4-μm thickness were prepared for pathological examination using standard hematoxylin and eosin (H&E) staining (Servicebio, China). The degree of hood fibrosis was assessed using Masson’s trichrome staining and Sirius Red staining (both from Servicebio, China). Bright-field images were acquired using a Pannoramic DESK digital slide-scanner (3DHISTECH, Budapest, Hungary) with Caseviewer 2.4 software. To further evaluate the extent of hood fibrosis, immunohistochemistry was conducted according to Zhong et al.’s protocol [70]. Immunofluorescence assays for PCNA were performed using anti-PCNA mouse monoclonal antibody (Servicebio, China) as previously described [71], with cell nuclei counterstained by DAPI (4′,6-diamidino-2-phenylindole). Total RNA was extracted from the entire hood tissue and then reverse-transcribed into cDNA for PCR amplification. The primers are listed in Table S6. Probe synthesis, sample treatment, and in situ RNA hybridization were performed as previously described [72]. Images were captured and analyzed under a confocal microscope (NOL-LSM 710 Carl Zeiss, Analytical and Testing Center, Institute of Hydrobiology, Chinese Academy of Sciences, Wuhan, China).

### TEM, x-ray microtomography, and 3-dimensional reconstructions

TEM was performed in accordance with the protocols established by Wu et al. [73]. The procedure included fixation of hood samples in 2.5% glutaraldehyde and 1% osmium acid solution, respectively, followed by dehydration, embedding, and sectioning to achieve 70- to 90-nm thin sections. These sections were then stained with lead citrate and examined using an HT7700 TEM (Analytical and Testing Center, IHB, CAS).

Microcomputed tomography (micro-CT) scans were conducted using a Skyscan 1276 scanner (Analytical and Testing Center, IHB, CAS), under conditions detailed by Gan et al. [74]. The resulting scan data were processed and rendered in AVIZO 3D v9.5.0 (Thermo Fisher Scientific), following the methodology outlined by Yi et al. [75].

### Measurement of ALP and TRAP enzymatic activity

The ALP and TRAP enzymatic activity was detected using Alkaline Phosphatase Assay Kit (Beyotime Biotechnology) and Tartrate Resistant Acid Phosphatase Assay Kit (Beyotime Biotechnology), respectively.

### Dual-luciferase assays

For eukaryotic expression, we constructed expression plasmids encoding goldfish proteins Rela-A (amino acids 1 to 591), June-A (amino acids 1 to 334), Cebpb-A (amino acids 1 to 277), Cebpb-B (amino acids 1 to 279), and Creb3l1-A (amino acids 1 to 405) by inserting the full-length open reading frames (ORFs) or corresponding DNA fragments into the pcDNA3.1(+) vector. Concurrently, we developed 3 promoter-driven luciferase reporter vectors, pGL3-col1a1b-A-pro-luc, pGL3-col2a1b-B-pro-luc, and pGL3-col5a3a-B-pro-luc. The plasmid construction and transfection protocols for dual luciferase adhered to methods detailed in previous studies [76–78]. DNA sequencing was employed to verify all plasmid constructs. Primers, including the sequences for restriction enzyme sites used in plasmid construction, are detailed in Table S6.

### Cytological experiments in vitro and ex vivo, and injection experiment in hoods

Primary hood cells were isolated from a healthy Lionhead goldfish and cultured under conditions detailed by Jiang et al. [79]. GICS cells were obtained from gibel carp (*Carassius gibelio*) skin tissue [79]. Ex vivo hood tissue explants, excised from live Lionhead goldfish, were maintained in L15 medium. Similarly, prior to qPCR, all 3 cell types/tissue explants were exposed to different concentrations of recombinant TNFα protein for 24 h. PA (200 and 400 mg/kg), AA (150 mg/kg), lipopolysaccharide (LPS) (20 mg/kg), and nigericin (4 mg/kg) were each dissolved in dimethyl sulfoxide (DMSO) and directly injected into the hoods of live Lionhead goldfish once a day at 40 μl per injection. Samples were collected for qPCR after 3 d of injection. PA (50 mg/kg) was also injected every 4 d at 40 μl per injection for 40 d for sectioning and in situ hybridization.

### Quantitative PCR

Hood tissues from WT crucian carp and Lionhead goldfish at developmental stages of 30, 50, 80, and 155 dpf (*n* = 3) were utilized for qPCR as previously described [17]. Upon reaching 90% confluence, Gibel Carp Brain cell line (GICB) cells were treated with LPS (200 ng/ml) for 3 h. Subsequently, the cells were incubated with AA (10 μM) and nigericin (5 μM) for an additional 2 h. Cells were then collected for RNA extraction and subsequent qPCR analysis. Additionally, primary fibroblasts were treated with recombinant prokaryotic TNF-α protein (synthesized by Genecreate, China) for 12 h. Post-treatment, these cells were harvested, and RNA was extracted and reverse-transcribed for qPCR analysis.

### Statistical analysis

Statistical analysis was conducted using GraphPad Prism 9.5. Error bars in the figures represent the mean ± SEM as indicated. The Student’s 2-tailed unpaired *t* test with 95% confidence was primarily used to determine significance, unless otherwise specified. Significance levels are denoted as *P < 0.05 and **P < 0.01.

### Animal works

This research work does not contain any studies with human subjects. All the animal experiments in this study were approved by the Animal Care Committee of Institute of Hydrobiology, Chinese Academy of Sciences (approval number “IHB-LL-2020002”).

## Data Availability

The data supporting the findings of this study are available from the corresponding authors upon reasonable request.
