# Supplementary material for: Arachidonic Acid Metabolism Down-Regulation-Mediated Tumor Necrosis Factor Signaling Contributes to Cutaneous Fibrosis and Skull Hyperplasia in Goldfish Hoods
Source: Research (Wash D C). 2025 Aug 6;8:0786. doi: 10.34133/research.0786 (PMC12327030; doi:10.34133/research.0786)
Supplement: Supplementary 1 — Figs. S1 to S4 Tables S1 to S6 [file research.0786.f1.zip › Supplementary Figures.docx]

**
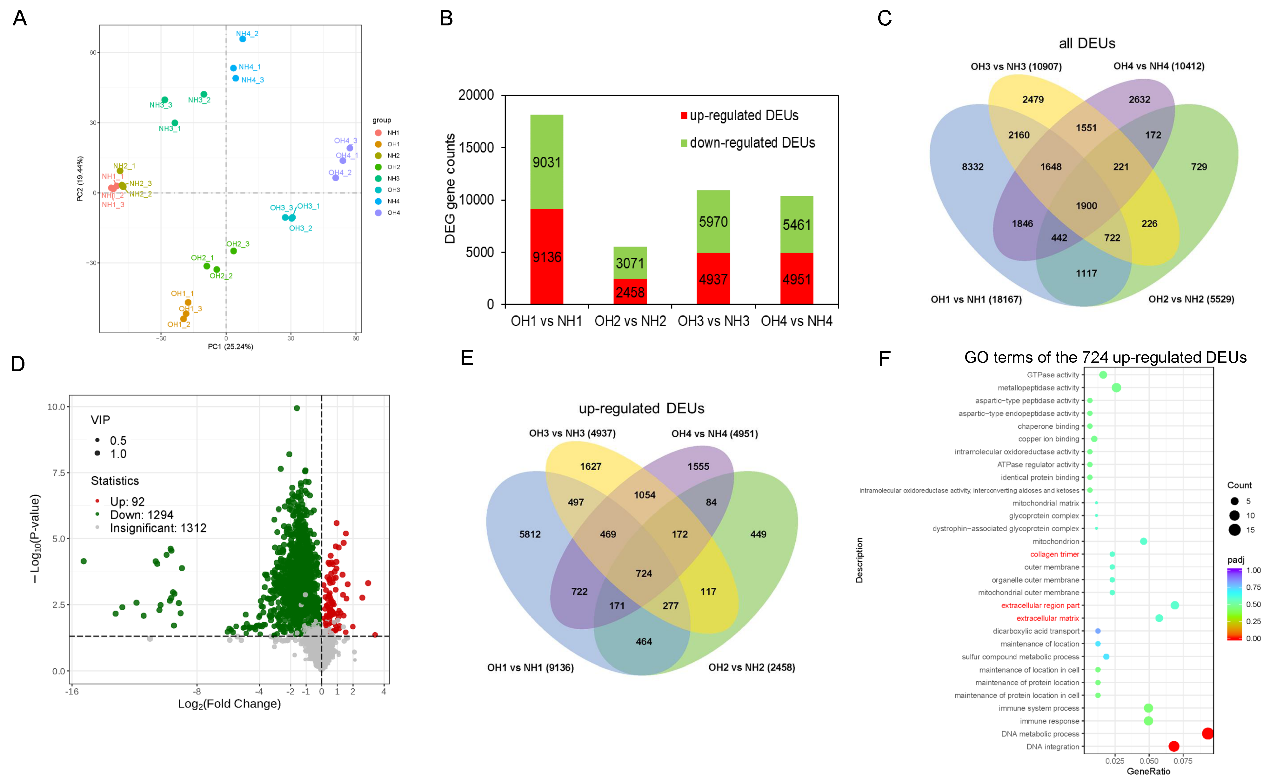
**

**Figure S1. Integrated analysis of differentially expressed unigenes (DEUs) and differentially accumulated metabolites (DAMs) across developmental stages.** (A) Principal component analysis (PCA) of transcriptomic data showing distinct clustering between normal head skin (NH) and oranda hood (OH) groups. (B) Bar plot displaying the total number of upregulated (red) and downregulated (blue) DEUs identified in pairwise comparisons. (C) Venn diagram illustrating the overlap of DEUs across four developmental stages (30, 50, 80, and 155 dpf). (D) Volcano plot of DAMs, with thresholds set at VIP > 1 and a p value < 0.05. Red and green dots represent significantly upregulated and downregulated metabolites, respectively. (E) Venn diagram of upregulated DEUs shared across all stages.

(F) Gene Ontology (GO) enrichment analysis of 724 consistently upregulated DEUs throughout the four periods.


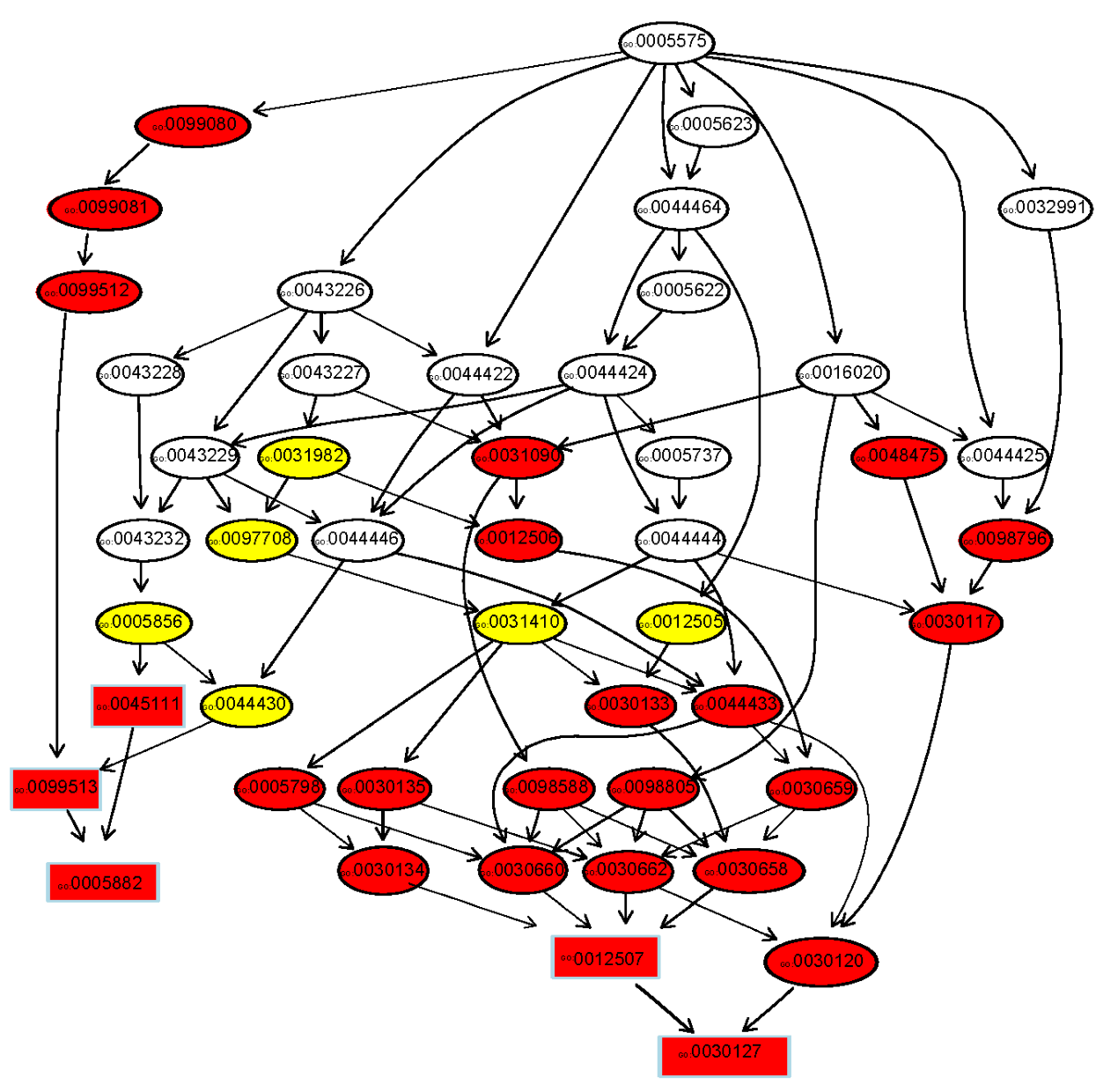


**Figure S2.** GO enrichment analysis of 1,900 DEUs consistently dysregulated throughout the four periods.


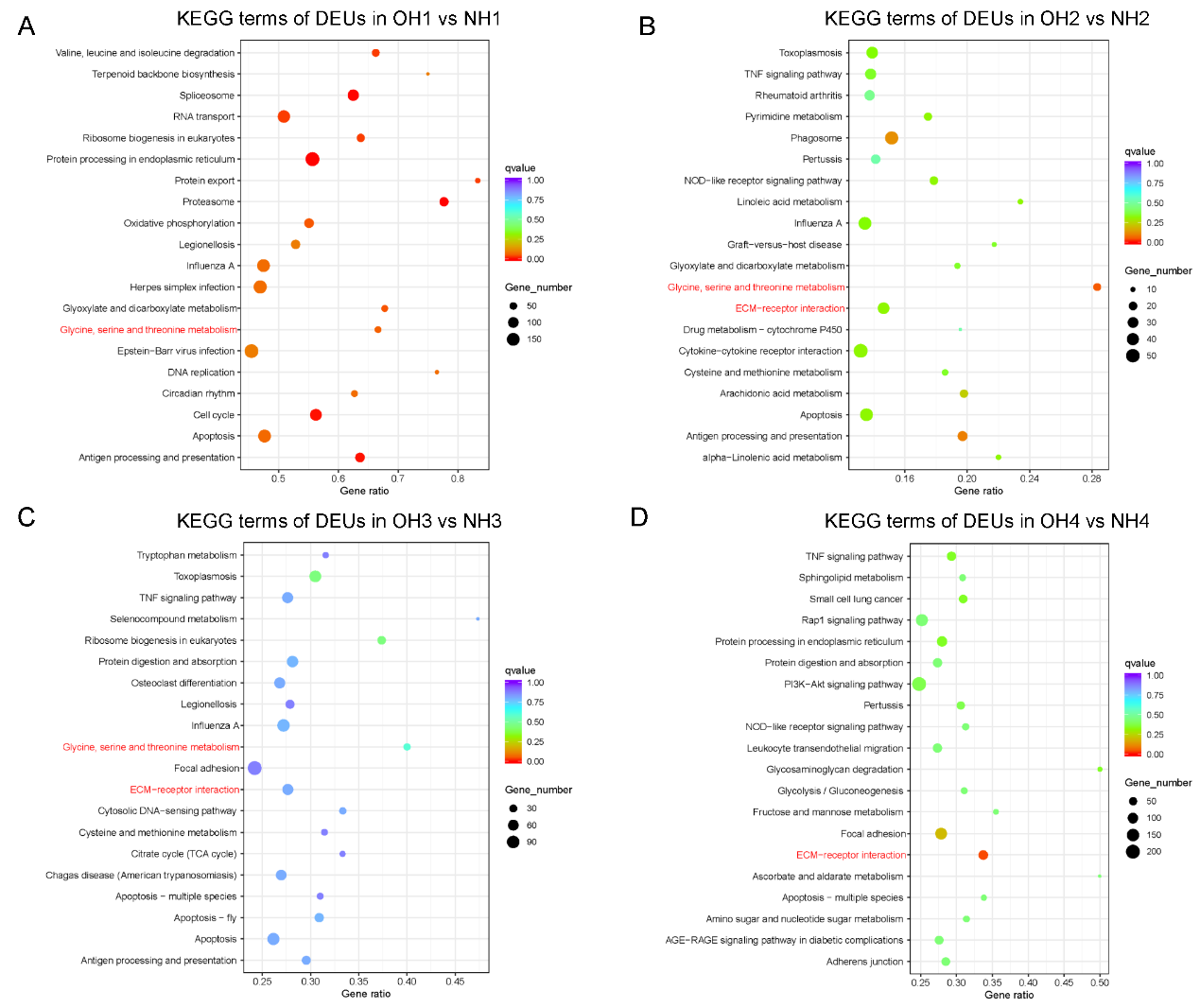


**Figure S3**. **Top 20 enriched KEGG pathways of the common DEUs among four development stages of 30 dpf (A), 50 dpf (B), 80 dpf (C), and 155 dpf (D), respectively.** The x-axis indicates the gene ratio of each pathway and y-axis shows pathway. The color and size of dot indicates Q value and the numbers of DEUs assigned to the corresponding pathway, respectively.


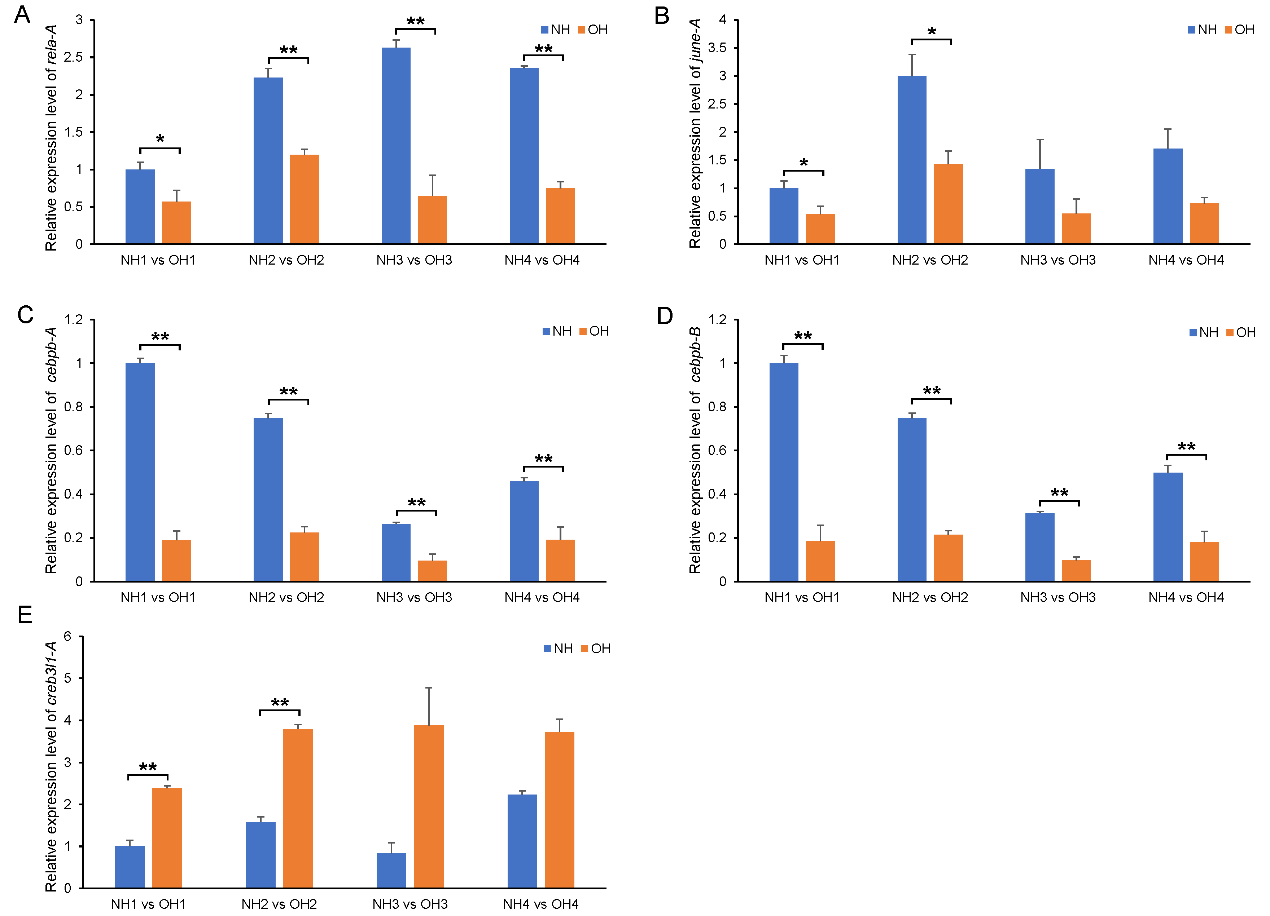


**Figure S4. Validation of transcription factor expression by qPCR.**

Relative mRNA levels of rela-A, june-A, cebpb-A, cebpb-B, and creb3l1-A in NH and OH tissues at four developmental stages. Data were normalized to the expression of normal hood at 30 dpf and expressed as mean fold change ± SD (n = 3). Significant differences between groups are indicated (**p <0.01, *p <0.05).
